# Towards Anatomic Scale Agent-Based Modeling with a Massively Parallel Spatially Explicit General-Purpose Model of Enteric Tissue (SEGMEnT_HPC)

**DOI:** 10.1371/journal.pone.0122192

**Published:** 2015-03-25

**Authors:** Robert Chase Cockrell, Scott Christley, Eugene Chang, Gary An

**Affiliations:** 1 Department of Surgery, University of Chicago, Chicago, IL, United States of America; 2 Department of Medicine, University of Chicago, Chicago, IL, United States of America; National Research Council of Italy (CNR), ITALY

## Abstract

Perhaps the greatest challenge currently facing the biomedical research community is the ability to integrate highly detailed cellular and molecular mechanisms to represent clinical disease states as a pathway to engineer effective therapeutics. This is particularly evident in the representation of organ-level pathophysiology in terms of abnormal tissue structure, which, through histology, remains a mainstay in disease diagnosis and staging. As such, being able to generate anatomic scale simulations is a highly desirable goal. While computational limitations have previously constrained the size and scope of multi-scale computational models, advances in the capacity and availability of high-performance computing (HPC) resources have greatly expanded the ability of computational models of biological systems to achieve anatomic, clinically relevant scale. Diseases of the intestinal tract are exemplary examples of pathophysiological processes that manifest at multiple scales of spatial resolution, with structural abnormalities present at the microscopic, macroscopic and organ-levels. In this paper, we describe a novel, massively parallel computational model of the gut, the Spatially Explicitly General-purpose Model of Enteric Tissue_HPC (SEGMEnT_HPC), which extends an existing model of the gut epithelium, SEGMEnT, in order to create cell-for-cell anatomic scale simulations. We present an example implementation of SEGMEnT_HPC that simulates the pathogenesis of ileal pouchitis, and important clinical entity that affects patients following remedial surgery for ulcerative colitis.

## Introduction

Technological advances in experimental procedures and data acquisition have lead to an unprecedented ability to examine the behavior of biological systems. The proliferation of “big data” in the biological sciences has significantly increased our ability to sort through the output of those experimental systems, and provides insight into the patterns that characterize the multiple different phenotypes associated with health and disease. However, the correlative nature of “big data” inherently limits the degree to which it can advance our understanding of how biological systems dynamically function at a fundamental level, which is a critical step in the development of effective means of controlling those systems. Adding to this problem is the fact that although we have access to more data than ever, that data is often too broad (per sample) while at the same time being too sparse (number of time points sampled). This leads to a condition were the field of systems biology is still “data poor” in terms of being able to inform characterization of the dynamical behavior of biological systems. Though there is not a lack of descriptive data, the temporal density of the data collected to date is not sufficient to determine mechanistic relationships in biological metabolic or gene-regulatory networks.

Dynamic computational modeling can help to bridge this gap by instantiating conceptual hypotheses of mechanistic relationships between biological objects of interest [1]. One method for dynamic computational modeling and simulation that is increasingly used to represent and investigate multi-scale biological systems is agent-based modeling [1–3]. Agent-based modeling is an object-oriented, discrete-event, rule-based, spatially-explicit, stochastic modeling method that maps well to biology: its conceptual basis of “things doing things” with an inherent ability to capture space/geometry/structure facilitates the ability of biomedical researchers to express and represent their hypotheses in an agent-based model (ABM) [4]. This is particularly true when cells are chosen as the agent level, and correspondingly there is an increasing use of ABMs to examine cell-population behaviors in tumor growth [5], wound healing [6], infection progression [7], and organ function [8].

However, the computational demands of large-scale ABMs are readily apparent. ABMs are computationally intensive compared to other modeling methods, such as differential equations: much like in reality, each simulated cell stores information regarding its function and current state. In biological cells, this information is stored in the form of DNA or RNA, whereas cells *in silico* store the information in data structures. As these models approach an anatomic scale, billions of cells must be simulated simultaneously. This becomes problematic for two major reasons: cycling through billions of cells on a single processor can take a significant amount of time, and anatomic scale simulations require large amounts of system memory.

In general, the behavior of every agent comprising an ABM is determined by an algorithmic rule set that can be efficiently executed on a single processor; the computational demands of an ABM primarily arise as the number of agents within an ABM increase. As such, ABM’s make appealing candidates for parallelization. The past ten years have seen parallel high-performance computing (HPC) platforms grow from thousands of processing cores to hundreds of thousands of processing cores, while the rate of floating-point operations per second (FLOPS) have increased by a factor of 1000 [9]. The rapid expansion of HPC capabilities coupled with the desire to create larger and more detailed ABMs has led to the development of ABM platforms that can take advantage of the distributed architectures present in modern HPC environments. The degree of agent-agent and agent-local environment interactions can challenge the parallelization of an ABM, and there have been several specialized modeling platforms developed to provide this capability: Biocellion [10,11], FLAME [12,13], and Repast HPC [14,15]. However, despite their advantages in terms of lowering barriers to use and implementation on HPC environments, there are potential limitations on the efficiency by which they can scale to anatomic-scale simulations(see Methods and Discussion). We believe that for specific organ system models, purpose built and optimized HPC ABMs may be beneficial in bridging cellular mechanisms to organ-level clinical pathophysiology.

Therefore, herein, we present an HPC implementation of our previously developed virtual gut models, the Spatially Explicit General-purpose Model of Enteric Tissue (SEGMEnT) [8], that demonstrates both its ability to simulate an anatomic scale clinical entity, illeal pouchitis following remedial surgery for ulcerative colitis, and its scalability to peta-scale/exa-scale HPC environments for full organ-level implementation.

## Methods

We have previously developed the Spatially Explicit General-purpose Model of Enteric Tissue (SEGMEnT), to dynamically represent existing knowledge of the behavior of enteric epithelial tissue as influenced by inflammation with the ability to generate a variety of pathophysiological processes. SEGMEnT [8] is a cell-level ABM: an object-oriented, discrete event, rule-based computational modeling method consisting of populations of computational entities (agents) that follow programmed rules governing their behavior with respect to the environment and interactions with other agents [2,3,16–19]. SEGMEnT dynamically represents and integrates existing knowledge concerning homeostasis and inflammation in the ileum and provides a computational platform to augment the exploration of the cellular/molecular processes involved in intestinal wound repair, ischemia/reperfusion injury, and colonic metaplasia/pouchitis. SEGMEnT successfully replicates the dynamically stable morphology and cellular populations of the healthy ileum, while qualitatively matching the spatial distribution of molecular signaling gradients consisting with the existing qualitative histological criteria utilized and reported in the literature [20–27]. SEGMEnT also reproduces the effects of inhibition of various signaling pathways by successfully representing the resulting phenotypes in terms of alterations of the crypt-villus architecture. With the integration of inflammation as a control input to the morphogenic signaling pathway, SEGMEnT demonstrates the ability to withstand certain acute perturbations (local tissue injury, ischemia/reperfusion) as would be expected from normal intestinal tissue, as well as reproduce a specific chronic pathological state associated with inflammatory stimulation (colonic metaplasia).

**Fig 1:**
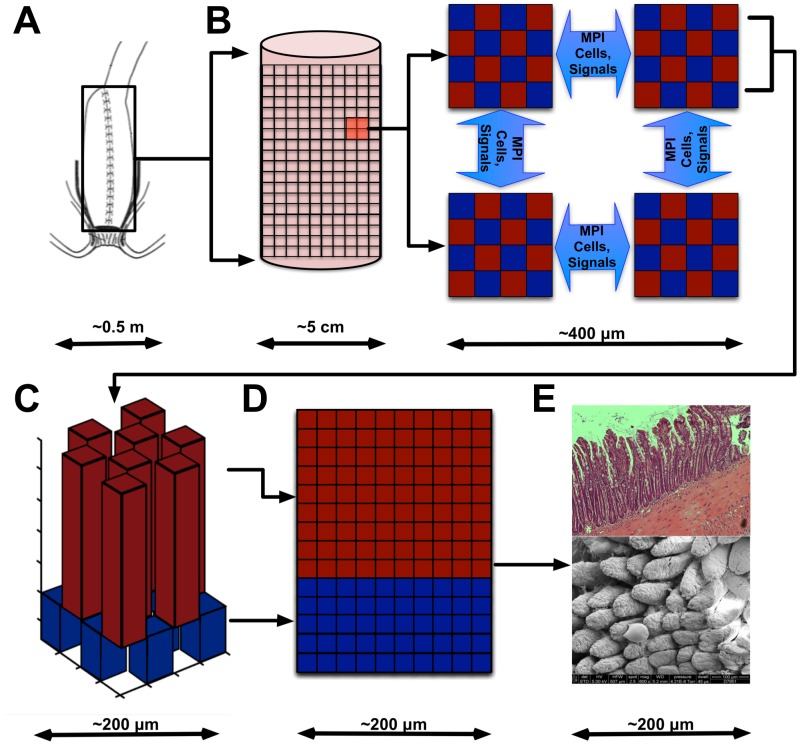
Panel A presents an illustration of anatomic configuration of the ileal “J-pouch.” Panel B illustrates how the j-pouch is modeled as a cylinder and distributed to the computational processes. Panel C shows how crypt and villus topologies are represented on a single processor. Panel D shows how these topologies are unwrapped to form a series of two-dimensional grids. This panel shows a congruent surface of a single face of a villus-crypt complex, where the villus component is shaded in red, and the crypt component shaded in blue. Note that the “central” portions of the villi and crypts are offset, leading to an alternating pattern of rectangular prisms formed by the planes of the two-dimensional grids. The portions of the grids that make up the tip of the villus and the valley of the crypt are omitted from this depiction. Finally, Panel E displays images of the actual biological system represented by SEGMEnT_HPC, seen in cross-sectional standard histology above and via scanning electron microscopy below.

SEGMEnT_HPC is a custom implementation of the model described in [8], and was developed in the C++ programming language. SEGMEnT_HPC utilizes the MPI 2.0 standard to perform inter-processor communications. Development of a custom ABM implementation for SEGMEnT_HPC offers us several advantages over using one of the existing modeling packages:

1. The tissue topology represented in SEGMEnT_HPC is both spatially complex and dynamic. Rather than using a single finite and continuous grid to represent the tissue, as is typical using ABM packages mentioned above, SEGMEnT_HPC uses a set of 2D grids which are then folded to create a tissue surface that represents a full three-dimensional histology (see Fig. 1). This then requires only a small fraction of the computational resources that would be required to maintain a full 3D grid. Additionally, the folded grids used in SEGMEnT _HPC are allowed to grow and shrink to match changes in the cellular agent populations during the course of a simulation run.
2. A custom implementation allows complete control over when in the simulation computation and inter-processor communication can occur. SEGMEnT_HPC uses several non-blocking MPI communication subroutines in order to overlap communication and computation and give SEGMEnT_HPC desirable scaling properties when moving to larger systems.
3. A customized implementation of SEGMEnT_HPC reduces the computational overhead that can be required by general-purpose ABM packages. In order to make their modeling framework sufficiently usable and generalizable for a wide variety of applications, general-purpose ABM package have overhead computation and memory costs that are not necessary for every simulation model; there is essentially a trade off between modeling flexibility and computational efficiency. A custom implementation of a specific model can avoid these additional costs. This is especially important when executing a simulation on tens to hundreds of thousands of processing cores as, at this scale, resources are limited by both monetary considerations and hardware availability.

**Fig 2:**
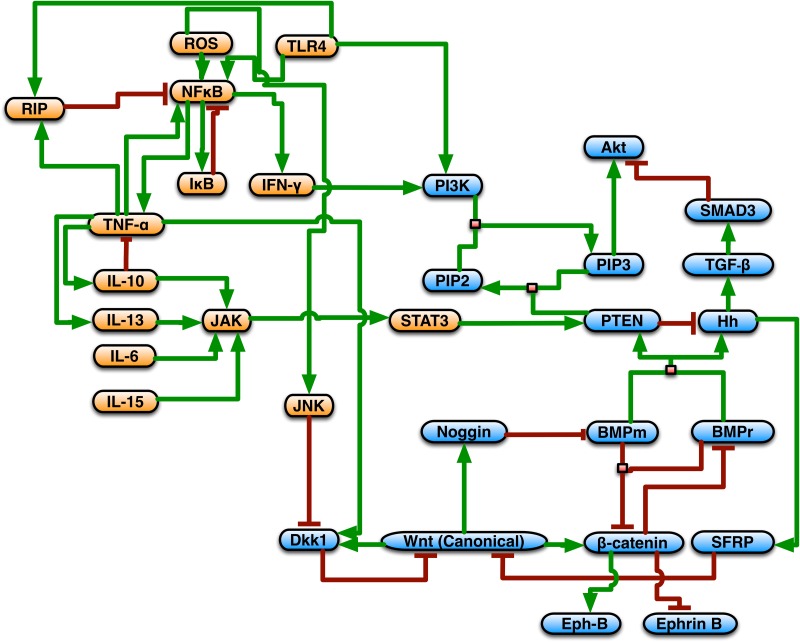
Signaling networks instantiated in SEGMEnT. Morphogen signaling pathway components are shaded in blue; inflammatory signaling components are shaded in orange. Stimulation/production relationships are depicted by green connectors; inhibitory relationships are seen as red connectors. The signaling network comprises the Wingless-related integration site (Wnt), Bone Morphogenetic Protein (BMP), Phosphotase and tensin homolog/phosphoinositide 3-kinase (PTEN/PI3K), Sonic Hedgehog Homolog (Hh), Tumor Necrosis Factor (TNF)-α, Interferon (IFN)-γ, RIP Kinase, nuclear factor kappa-light-chain-enhancer of activated B cells (NF-κB), Janus Kinase (JAK), Signal transducer and activator of transcription 3 (Stat3), and reactive oxygen species (ROSs), and Interleukin (IL) 6,10,13, and 15 signaling pathways.

In order to parallelize the model, the area of tissue to be simulated is spatially discretized such that each processing core involved in the simulation has control of one of these congruent sections (See Fig. 1). The progression from Panel A to Panel E depicts the successive relationships between the anatomic configuration of the ileal “J-pouch” (Panel A), how the j-pouch is modeled as a cylinder and distributed to the computational processes (Panel B), how crypt and villus topologies are represented on a single processor (Panel C), how these topologies are unwrapped to form a series of two-dimensional grids (Panel D), and finally the relationship between the ABM topology and images of the intestinal surface structure (Panel E). Individual crypts and villi contain an set of grids which store the cellular agents comprising the simulation as well as relative concentrations of the various signaling molecules implemented in SEGMEnT_HPC (See Fig. 2). Crypts and villi that are located on the spatial processor boundary contain an additional buffer in order to maintain continuity with neighboring processors. The buffer extends all grids by one cell length in order to synchronize cellular motion and chemokine diffusion across processes. This buffer is updated via MPI communication at each time step.

Parallel simulations were performed on the Beagle and Midway supercomputers at the Computation Institute and Research Computing Center respectively at the University of Chicago. Beagle has 726 Non-Uniform Memory Architecture nodes; each node has two 12-core AMD Opteron 6100 series processors with 1.5 GB of RAM per processing core. Midway has 284 tightly coupled compute notes; each node has two 8-core 2.6 GHz Intel Xeon E5-2670 processors with 2 GB of RAM per processing core.

### Methods–Validation

SEGMEnT_HPC is a high-performance computing, distributed extension of the previously developed SEGMEnT [8]. Validation of the initial SEGMEnT model involved the replication of baseline healthy ileal tissue dynamics by generating realistic cellular populations with topological tissue features matching known histology in a dynamically stable steady state. Simulated chemokine gradients, including Wingless-related integration site (Wnt), Bone Morphogenetic Protein (BMP), Sonic Hedgehog Homolog (Hh), and Protein Kinase B (Akt), match those reported in the literature both when simulating healthy tissue and a selection of gene knockout experiments (Wnt, Hh, and PTEN). Validation simulations for SEGMEnT_HPC used these same criteria, and produced similarly recognizable behaviors at similar scale representation compared to the original SEGMEnT. The more macro-scale behavior of the distributed SEGMEnT_HPC was evaluated in a qualitative fashion through the generation of biologically plausible tissue defects in representation of a larger section of intestinal tissue.

### Methods–Biological Background and Motivation

Inflammation of the illeal pouch following remedial surgery for ulcerative colitis, a condition termed pouchitis, is a significant source of morbidity in patients with inflammatory bowel disease with reported long-term incidence rates of up to 95% [28]. The pathogenesis of pouchitis is believed to involve the intersection of dysregulated intestinal inflammation, abnormal mucosal tissue response, and alterations in gut microflora due to stasis resulting from the anatomic configuration of the illeal pouch. Thus the pathogenesis of pouchitis is a multiscale process that extends from microscale molecular signaling to tissue scale cellular patterning to anatomic-scale dynamics of the flow of intestinal contents. Given that the progression of pouchitis and the spatial distribution of the stimuli that drive it are not homogenous throughout the entirety of the pouch, anatomic scale simulations are required in order to plausibly simulate the complicated interplay of ileal and rectal (colonic) tissue with a dynamic microbiome.

The fecal microbiome is modeled as a slow-flowing liquid that fills up the ileal pouch in approximately 4 hours. New stool enters the pouch at 15 minute intervals as this is the length of a time step in SEGMEnT_HPC, and the flow is modeled indirectly rather than using computationally expensive fluid dynamics calculations. This approximation is sufficient for SEGMEnT_HPC as the only importance that stool currently has its contact with the epithelial tissue. At simulated 4-hour intervals, the ileal pouch is emptied and the stool once again begins to fill the pouch. The fecal microbiome interacts with the epithelium through Toll-Like Receptor (TLR) signaling (see Fig. 2). The strength of TLR activation is given by an “inflammatory potential” which increases linearly over time, such that:

$${IP}_{t}\;=\;{IP}_{t-1}+{∆}_{IP}$$

This increase of inflammatory potential over time is a necessary condition for fecal stasis to cause inflammation-induced metaplasia in the ileal pouch. In a state of constant flow, the pouch and the entirety of the small intestine would constantly be exposed to fecal matter, whether or not it is static, however this metaplasia occurs in the pouch, not in the proximal intestine leading into the pouch [29,30]; thus, the metaplasia forms a gradient, with the most significant metaplastic changes occurring in the terminal ileal pouch.

## Results

### Results–Scaling

**Fig 3:**
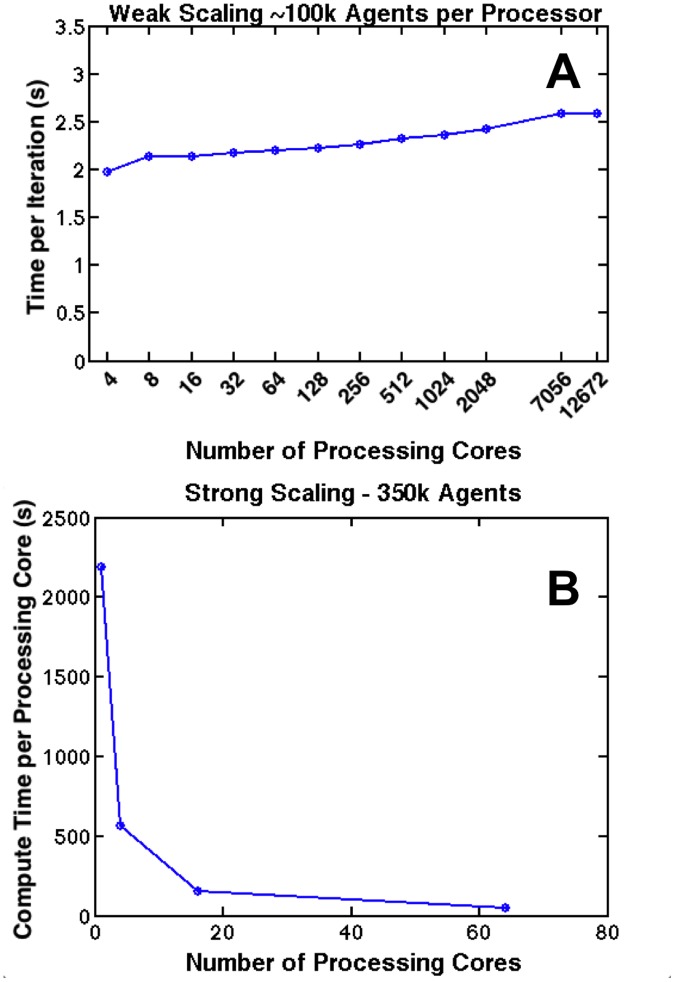
Scaling Curves. Panel A displays the weak scaling curve for SEGMEnT_HPC. Compute time per iteration is plotted against the number of processing cores simultaneously simulating ~100,000 cells. Panel B displays the strong scaling curve for SEGMEnT_HPC. Compute time per processor is shown for ~350,000 agents on 1, 4, 16, and 64 processing cores. Each processing core simulates 256, 64, 16, and 4 crypt-villus topologies respectively.

**Fig 4:**
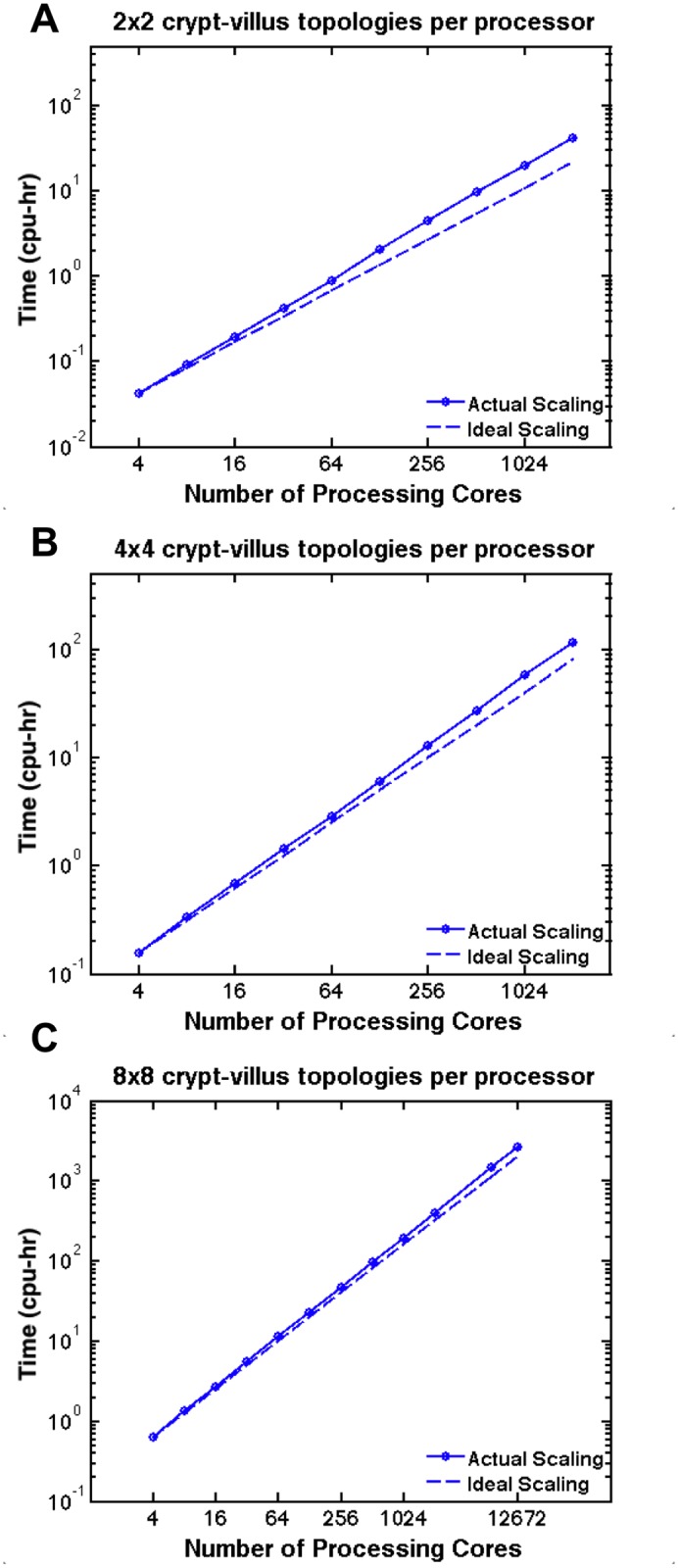
Tissue Scaling Curves. We present tissue scaling curves for systems of 4, 16, and 64 crypt-villus topologies. The number of processors simulating these topologies is plotted against the total CPU-time utilized by the simulation. As the number of topologies per processor is increased, SEGMEnT_HPC approaches the ideal scaling curve. The maximum number of processors tested was 12,672. SEGMEnT_HPC displays excellent potential to scale up to anatomic scale (>100,000 processing cores) while making efficient use of computational resources, as there is no evidence of significant divergence between the actual and ideal scaling curves.

In Fig. 3, we present examples of SEGMEnT_HPC’s weak (Panel A) and strong (Panel B) scaling curves. The weak scaling curve shows how SEGMEnT_HPC scales as we add tissue area to the simulation. As we increase the size of the simulation from 4 to 12,672 processing cores, the average time per model iteration increases by approximately 25%; however, as we approach 10,000 processing cores, the weak scaling curve begins to level off, suggesting that SEGMEnT_HPC will scale well as we continue to increase the size of the simulation. As we partition the simulation spatially, each processor only communicates with its nearest neighbors; we expect that this framework will avoid congestion of the communication network and results in the leveling off shown at 7056 processing cores in Fig. 3, Panel A. The strong scaling curve (Fig. 3, Panel B) shows how SEGMEnT_HPC scales when we distribute the same sized problem over an increasing number of processors. The efficiency of the simulation decreases to 90% as we move from 1 to 16 processing cores. It should be noted that this not the type of parallelization that SEGMEnT_HPC is intended to provide. As we move towards anatomic scale simulations, it will be necessary to maximize memory utilized per processing core. As such, we present alternative scaling curves in Fig. 4. Moving from left to right, Fig. 4 demonstrates how SEGMEnT_HPC scales when simulating increasing areas of tissue on an increasing number of processing cores on systems containing 4, 16, and 64 crypt-villus topologies. It is clear that SEGMEnT_HPC scales best when the maximum number of agents is assigned to a processing core; this is to be expected as maximum utilization of available memory also minimizes the overall ratio of inter-process communication to computation. As we move from 1 to 12,672 processing cores, the compute time per processor increases only by a factor of 1.3.

### Results–Sample Output

**Fig 5:**
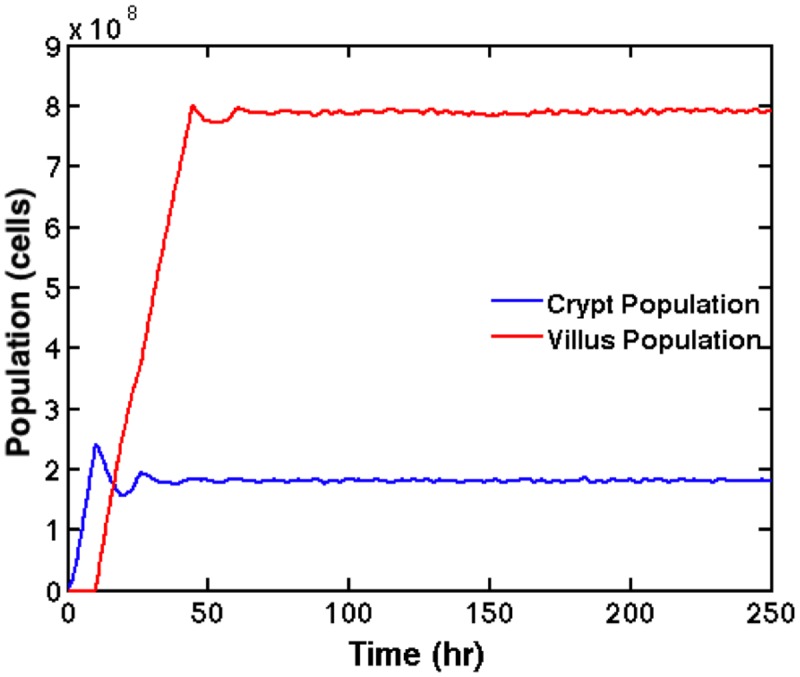
Healthy Tissue Cell Population. Total cellular population for a simulation of healthy epithelial tissue on 12,672 processing cores is plotted against simulated time. At peak population, 1,038,136,974 cells were simulated simultaneously. The time period prior to equilibrium is an initialization stage for the model. Note the initial rise in cellular populations and subsequent short oscillation are artifacts due to initialization and equilibration of the simulation.

**Fig 6:**
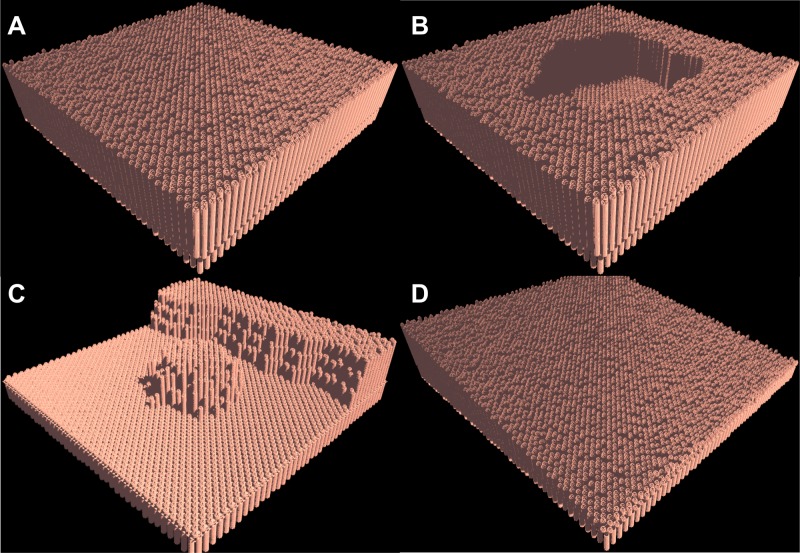
Tissue Rendering. Fig. 6 displays 16 mm^2^ tissue sample cutouts (Panels A, B, and C) and a 20 mm^2^ cutout (Panel D). These renderings are a post-processing output of SEGMEnT_HPC. Cellular spatial location information for select processing cores is written to file. These coordinates are then mapped from their two-dimensional grids back onto the topology that they represent. The entire simulation is not rendered due to size. Panel A presents healthy tissue in homeostasis. Panel B presents tissue with an applied circular ulcer, which spreads slightly due to inflammation. Panel C presents tissue recovery after inflammatory stimuli has been removed. Panel D presents tissue that has been exposed to an inflammatory gradient as described in the sample output section above.

Fig. 5 displays the total cellular population over simulated time for a healthy tissue sample sized 1,129 mm^2^ simulated over 7056 processors; the ratio of villus population to crypt population is approximately 4:1, which matches histology, both in terms of crypt/villus feature sizes, and in terms of absolute populations [27]. Fig. 6 displays 16 mm^2^ cutouts from a healthy tissue sample (Panel A), tissue with an applied ulcer (Panel B), and tissue recovering from severe inflammation (Panel C), and a diffuse reducing of villus height seen in pouch metaplasia (Panel D).

Pouch metaplasia simulations were performed on 12,672 processing cores The simulated pouch had a diameter of 3.5 cm and a length of 6 cm, which is approximately ½ of anatomic scale [31]. Inflammatory potential of the stool was calibrated such that it maximized at the value necessary to induce the metaplasia demonstrated in [8]. Fig. 6, Panel D shows a 16 mm^2^ area of epithelial tissue from the above simulation of pouch metaplasia. This tissue section has been unwrapped such that it is presented as a rectangle.

## Discussion

The future of biomedical research will require the ability to effectively leverage the incredible advances in computing technology in order to more effectively exercise the biomedical task of developing more effective therapeutics. Intrinsic to this task is the ability to translate cellular and molecular mechanisms (invariably the targets for new drugs) into clinically relevant organ-level physiology and pathophysiology. Agent-based modeling has been increasingly utilized as a means of dynamically representing knowledge, with a specific benefit of explicitly being able to reproduce the spatial properties of biological systems/tissues/organs. A natural evolution of the use of ABMs is the eventual goal of being able to create cell-for-cell level representations of entire organs, i.e. anatomic scale ABMs. The potential impact of this goal is readily obvious, and would represent perhaps the ultimate ability to translate detailed molecular knowledge concerning the behavior of individual cells into virtual organ systems that produce clinically relevant physiology and pathophysiology, i.e. clinical disease phenotypes. However, the recognition of the scale of such an endeavor is daunting when one realizes the actual number of cells present in the primary organs of interest: the heart contains ~2–3 billion myocytes with ~10 billion overall cells, the liver contains ~150–200 billion hepatocytes, the brain over 400 billion cells and the intestinal tract over 500 billion cells. Even if the functional goal is not to create a cell-for-cell representation of an organ, it is clear to being able to comprehensively represent the range of heterogeneous cellular behaviors present at clinically-relevant anatomic scale will require the ability to develop and utilize ABMs incorporating billions of computational agents. An example of the need for anatomic-scale representation can be seen in the pathogenesis of intestinal disease, where there are multiple biological/organizational levels of clinically relevant system phenotypes. These include: cellular tissue patterning, which determines the histological diagnosis of pathology; loco-regional phenomena, including tumor growth and local, surgical wound healing, and organ-level distribution of disease as seen in inflammatory bowel disease, which has known regional patterns and progression. The generative processes for gastrointestinal disease, as with nearly all disease processes, are driven by microenvironmental factors operating on and intersecting with a range of host-side genetic potential. The regional nature of intestinal disease suggests that shifts in the heterogeneous distribution of microenvironments plays an important role in the pathogenesis of disease; in fact these shifting microenvironments are intrinsic to the function of the intestinal tract, which involves the transit of nutrients and waste contents throughout its length. This in turn suggests that the effective characterization of clinically relevant disease phenotypes needs to capture these multi-scale phase transitions that occur as micro-level cellular and molecular interactions cascade and manifest at anatomic scale.

However, being computationally intensive, the ability to effectively scale up ABMs to a desired anatomic scale requires utilizing the most advanced and powerful computing architectures. Several groups have developed general-purpose agent-based modeling toolkits to meet this need:

1. The Flexible Large-scale Agent Modeling Environment (FLAME) [12,13] has demonstrated the ability to parallelize ABMs consisting of hundreds of thousands of agents up to hundreds of processors [12]. FLAME represents agents using Communicating Stream X-Machines [32] in which agents are defined through a set of state transition functions. In aggregate, these transition functions form an acyclic state machine. Agents communicate via message boards such that the model is fundamentally parallel. FLAME is a multi-purpose ABM framework and can be used for a wide range of models, ranging from biological simulations [33] to large scale economic models [34].
2. Repast HPC [15] is a parallelized ABM framework developed at Argonne national laboratory which has been primarily used for social science simulations [35], though it has the ability to perform biological simulations as well. Scaling studies have been performed on Repast HPC using up to 32,678 processing cores.
3. Biocellion [10,11] is a ABM framework designed for simulating molecular and cellular dynamics which has reported benchmarks on up to 4096 processing cores. Biocellion provides a template for modeling physical forces and interactions between cells in much greater detail than alternative frameworks, making it ideally suited to simulate morphogenetic processes.

ABM toolkits, such as those mentioned above, lower the barrier to entry for researchers wishing to begin a modeling program and can assist in making developed models accessible outside of the group that created them; however these toolkits are not without drawbacks. When utilizing an ABM toolkit, one is limited to biological functions and features that the toolkit creators implemented. Additionally, when considering a parallel implementation, ABM toolkits typically will not scale as well as a custom implementation, especially as models become larger and require thousands to hundreds of thousands of processors. These scaling problems arise both from the overhead necessary to run a generalized modeling framework as well as a “one-size fits all” implementation of parallelization. Additionally, scaling studies are typically performed on simplified toy models, and do not necessarily give data for how the toolkit will perform when simulating a more complex system. For example, as SEGMEnT_HPC scales from 1 processing core to ~10,000 processing cores, each responsible for simulating ~100k agents, the compute time per processor only increases by a factor of 1.2. This benchmark represents actual performance of the model as in simulates larger and larger areas of tissue. As the approximate value of a CPU-hour is $0.10 [36], savings from using a custom simulation can become significant when running on tens to hundreds of thousands of processing cores.

SEGMEnT_HPC has demonstrated a clear potential to scale up to current petascale HPC platforms. Looking forward towards exascale computing, we estimate that SEGMEnT_HPC would require 450,000 processing cores for 30 hours (at the current temporal resolution) to simulate the healthy colon for 1 year. We estimate that it simulation of the small and large intestines together for 1 year would require approximately 2.4 million processing cores for 40 hours.

In order to plausibly simulate inflammatory bowel disease, additional cell types will have to be added to SEGMEnT. A module representing the adaptive immune system will be necessary to simulate inflammation on a time scale greater than a few days. We anticipate that this will incorporate multiple t-cell lineages as well as further characterization of the functional properties of the microbiome, including microbial population dynamics

In the longer term, we intend to be ready to perform whole-organ simulations when the exascale computing era arrives. In order to simulate long-term inflammation at anatomic scale, a dynamic load balancing system will be required in order to maximize the utility of our computational resources. Simulations of significant infection can double the number of agents simulated per area, causing load imbalances (provided at least some of the system remains in an un-inflamed state). Dealing with this issue at petascale (currently) or exascale (future) represents a significant software engineering challenge.
